# Development and characterisation of fast dispersible dimenhydrinate tablets: Compactional study and *in-silico* PBPK modeling

**DOI:** 10.1371/journal.pone.0334421

**Published:** 2025-10-27

**Authors:** Uroosa Maqbool, Farya Zafar, Riffat Yasmin, Huma Ali, Hira Akhtar, Rizwana Rehmat, Yumna Tahir, Iqra Haider, Shumaila Anwar, Javeria Ameer

**Affiliations:** 1 Department of Pharmaceutics, Faculty of Pharmacy and Pharmaceutical Sciences, University of Karachi, Karachi, Pakistan; 2 Department of Pharmaceutics, Faculty of Pharmacy, Dow College of Pharmacy, Dow University of Health Sciences, Karachi, Pakistan; 3 Institute of Pharmaceutical Sciences, Jinnah Sindh Medical University, Karachi, Pakistan; Vijaya Institute of Pharmaceutical Sciences for Women, INDIA

## Abstract

The aim of this study was to optimize and evaluate dimenhydrinate fast dispersible tablets (50 mg) by utilizing cost effective direct compression method. A total of nine formulations (F1-F9) were designed and developed by central composite rotatable design using design expert^®^ software (version 11.0, Stat-Ease Inc.,) to study the impact of avicel PH102 (15–55%) and sodium starch glycolate (2–8%) on responses, i.e., hardness (R_1_), disintegration time (R_2_) and % drug release (R_3_). Powder blends of all formulations showed good flow behavior. Post compressional studies were conducted to assess the quality attributes of the compressed formulations. Formulation F2 was found to be optimized with good mechanical strength, i.e., hardness 4.2 kg, friability 0.77%, disintegration time 19 secs and % drug release 100.01% at 15 minutes. Compressional behavior of optimized formulation was determined with the help of Heckle plot. The PY (yield value) and the tensile strength of the optimized formulation (F2) were found to be 66.66 MN/m^2^ and 1.093 ± 1.66 to 1.642 ± 1.76 MN/m^2^ respectively. Differential scanning calorimetric and scanning electron microscopy analysis were performed to explore compatibility between ingredients and morphological features. All formulations followed weibull model in four different dissolution media. All the formulations were found to be stable at accelerated conditions. The in-silico GastroPlus™ PBPK modeling was also carried out to determine the pharmacokinetic parameters of the optimized formulation (F2) as an alternative to *in vivo* dimenhydrinate studies. Simulated PK values of F2 were found to be 143.16 ng/ml (C_*max*_), 2 h (T_*max*_), 2533.8 ng-h/ml (AUC _0-inf_) and 1477.9 ng-h/ml (AUC _0-t_). Results indicated that the fold error value was > 2 which indicated that experimental values are comparable with the predicted values. This type of study will be helpful for pharmaceutical manufacturers to produce patient compliance, low-cost tablet dosage form.

## Introduction

Oral solid dosage form remains the most used method of drug administration and among them fast dispersible tablets gain more attention as these formulations offers patient compliance and ease of administration. So, these are mainly designed for pediatric and elderly patients having difficulty in swallowing. Conventional tablet formulations showed delayed absorption and late onset of action. To overcome these issues, fast dispersible tablets immediately release the drug from dosage form after rapid disintegration [1]. Dispersible tablets are of two types: one is directly disintegrated in the oral cavity without the use of water in saliva after administration and another one is rapidly disintegrated or dispersed in water prior taken up by the patients [2]. Oral and pre gastric absorption of API from this type of formulation reduces disintegration time (usually less than a minute) and avoiding first pass metabolism, increases the bioavailability of the drug than conventional tablets which is mainly due to the addition of super disintegrants [3].

Super disintegrant may be incorporated intra granularly, extra granularly or by the combination of both [4]. Dispersible tablets are manufactured by different techniques like lyophilization, sublimation, molding, hot melt extrusion, wet granulation and direct compression methods, but among them the most preferable method is direct compression due to convenience, high productivity, cost effectiveness, suitability for heat and moisture sensitive substances and its fundamentally continuous production nature [5].

Formulation designing and optimization is widely performed by central composite design (CCD) in pharmaceutical industries to select the right concentrations of chosen excipients and to evaluate the effects of independent variables on response variables to choose the best formulation [6]. Irrespective of tablets’ method of manufacturing, tablets must possess sufficient mechanical strength to bear the hazards of transportation, handling and storage [7]. Evaluating the compressibility of a tablets under mechanical stress is necessary part of pre-formulation study. Compressibility evaluation significantly contributes to achieve a science-based tablet formulation design which helps in controlling the entire manufacturing process following QbD (quality by design) approach [2]. The tablet compression involves different steps including die filling, particles rearrangement, particles fragmentation, plastic/elastic deformation of particles under applied mechanical stress [8]. All these mechanisms contribute in the creation of tensile strength of the tablets [9]. For the determination of relationship between compressional pressure and mechanical (tensile) strength of the compact, different researches have been reported; since application of stress naturally result in reduction of powder bed height or volume [7]. In this study Heckel analysis will be performed to explore the nature of deformation of powder blend after application of compressional pressure [2].

Dimenhydrinate belongs to biopharmaceutical classification system (BCS) Class II, it is the H_1_ receptor antagonist with anti-histaminic effect thus preventing nausea and vomiting. Its half-life is 3–9.3 hrs [10] so it is considered as an ideal candidate for designing the water dispersible formulation using super disintegrants in order to enhance its solubility and dissolution rate [11].

Physiological – Based Pharmacokinetics (PBPK) modeling and simulation is an *in silico* computational tool for the assessment and calculation of the *in vivo* presentation of the API based on the collected knowledge of the biopharmaceutics and pharmacokinetic profile of the given compound [12]. PBPK modeling and simulation is now becoming an integral part of drug discovery and delivery [13].

In the present study, fast dispersible dimenhydrinate 50 mg tablets were designed and optimized by central composite design (CCD) having variable concentrations of avicel PH102 and sodium starch glycolate. The estimated responses were hardness (R_1_), disintegration time (R_2_) and % drug release (R_3_). For the development of tablets direct compression method was selected. Pre-formulation study and post compression quality control tests were performed on all designed formulations and finally stability testing was accomplished for shelf life estimation following international committee on harmonization [14]. The Heckel analysis was also carried out on optimized formulation to determine the compaction behavior. Also PBPK modeling was applied to predict the pharmacokinetic parameters of the optimized formulation (F2).

## Experimental design

### Materials and methods

#### Chemicals and reagents.

Dimenhydrinate was gifted consummated by Searle Pharmaceuticals (Pvt.) Ltd., sodium starch glycolate, Avicel PH 102, magnesium stearate, aspartame (FMC Corporation^®^, USA), sodium, potassium dihydrogen phosphate, hydrochloric acid (Merck^®^, Darmstadt, Germany) and distilled water.

#### Apparatus and equipment.

Analytical weighing balance (EB-340MOC, Shimadzu), vernier caliper (Digital Caliper: Seiko brand), single punch compaction machine (Korsch Erweka, Frankfurt Germany), dott bonapace compression machine CPR 18 (Dott Bonapace & Co. Italy), hardness tester (Fujiwara Seisakusho, Ogawa Seiki Co Ltd, Tokyo, Japan), friabilator (H. Jurgens and Co- GmbH and CoD2800, Bermen, Germany), USP dissolution apparatus II (Paddle apparatus – II) (Erweka, DT 600, Husenstamm, Germany), climatic chamber (Nuaire) and UV spectrophotometer (UV-1800 Shimadzu Corporation Kyoto, Japan).

#### Soft wares used.

Design Expert^®^ version 11.0 (Stat-Ease, Inc, Minneapolis), Minitab^®^ 19.0., Microsoft Excel^®^2013, Microsoft Word^®^ 2013,DD solver^®^ and GastroPlus 9.8.3 (Simulations Plus, Inc., Lancaster, CA)

#### Formulation design.

Nine fast dispersible formulations of dimenhydrinate (50 mg) tablets were designed and optimized by central composite design (Design Expert^®^ version 11.0). The response of independent variables such as (X_1_) avicel PH102 (15−55%) and (X_2_) sodium starch glycolate (2−8%) [15] with five different levels (i.e., −1,1,0, **-**α, α) was observed on tablets’ parameters, i.e., hardness (R_1_), disintegration time (R_2_) and % drug release (R_3_) at 15 minutes. Other excipients such as magnesium stearate and aspartame were used at a fixed percentage (i.e., each 1%). Significant model was selected based on fit summary and ANOVA. Level of variables for optimization of formulations and composition of dimenhydrinate fast dispersible tablets are mentioned in (Table 1 and 2) respectively.

**Table 1:** Level of variables for optimization in tablet formulation.

VARIABLES	DESIGN MODEL	LEVEL OF VARIABLES				
		−1 (%)	+1 (%)	0 (%)	-α (%)	+α (%)
Avicel – PH 102 (**X**_**1**_)	QUADRATIC	15	55	35	6.72	63.284
Sodium starch glycolate (**X**_**2**_)		2	8	5	0.757	9.24

**Table 2:** Composition of Dimenhydrinate fast dispersible (50mg) tablets using Central Composite Design.

Run	Space Type	Avicel PH 102 % (mg)	Sodium Starch Glycolate % (mg)	Magnesium Stearate (mg)	Aspartame (mg)	API (mg)	Tablet weight (mg)
**F1**	Axial	63.284 (99.044)	5 (7.825)	1.565	1.565	50	160
**F2**	Center	35 (91.666)	5 (13.095)	2.619	2.619	50	160
**F3**	Factorial	15 (66)	8 (35.2)	4.4	4.4	50	160
**F4**	Factorial	55 (93.076)	8 (13.538)	1.692	1.692	50	160
**F5**	Axial	6.715 (53.860)	5 (40.099)	8.019	8.019	50	160
**F6**	Axial	35 (83.256)	9.242 (21.986)	2.378	2.378	50	160
**F7**	Factorial	55 (102.542)	2 (3.728)	1.864	1.864	50	160
**F8**	Axial	35 (101.966)	0.757 (2.206)	2.913	2.913	50	160
**F9**	Factorial	15 (86.842)	2 (11.578)	5.789	5.789	50	160

*F2 = Center point formulation.

#### Pre-compression studies.

**Evaluation of densities and flow properties of powder blend:** Bulk and tapped density of the powder blends were performed by tapping method (100 times) using graduated cylinder [15]. Bulk density and tapped density (gm/cm^3^) were determined by following equations:


$$p_{tapped}=\frac{Mass}{Bulk\;volume}$$ (1)

Whereas, tapped density (gm/cm^3^) was determined by following formula:


$$p_{tapped}=\;\frac{Mass\;}{Tapped\;volume}$$ (2)

Flow properties of powder blends were evaluated by following parameters [16]:


$$Carr^{\prime }s\;Index=\text{1}\text{0}\text{0}\times \left(\frac{Tapped\;bulk\;density-Poured\;bulk\;density}{Tapped\;bulk\;density}\right)$$ (3)

$$\text{ Hausne}{\text{r}}^{\prime }\text{s ratio}=\frac{\text{tapped density}}{\text{bulk density}}$$ (4)

For angle of repose, *fixed cone height* method was used. Following equation was used for the assessment of angle of repose [2]:


$$\theta ={tan}^{-\text{1}}\;\text{2}\;hr$$ (5)

Where, θ is the angle of repose, ‘h’ is the height of the heap and ‘r’ is the diameter of the heap formed.

**Compression of powder blends:** Direct compression method was used for manufacturing of fast dispersible dimenhydrinate (50 mg) formulations (F1-F9). Prior to passing through sieve of 20 mesh size, all ingredients (including API, avicel PH 102, sodium starch glycolate, magnesium stearate and aspartame) were weighed accurately and then mixing was done in a polybag for six minutes by tumbling action. Then magnesium stearate was added, and the blend was further mixed for 5 minutes. Tablets were compressed by using single punch tablet compression machine.

### Physicochemical evaluation of dimenhydrinate fast dispersible tablets

Compressed tablets were evaluated for weight variation test where no more than 2 tablets should be of different weight as compared to the average weight [16]. Diameter and thickness of compressed tablets were evaluated with the help of digital vernier caliper (Digital Caliper: Seiko brand), average diameter and thickness should be within ± 5% of standard value. Hardness tester (OSK Fujiwara, Ogawa Seiki Co. Ltd., Tokyo, Japan) was used for the determination of tablet hardness and the hardness of fast dispersible tablets should be in between 3–8 kg (Liu et al., 2002). Percentage friability was estimated with the help of Roche type Friabilator (H. Jurgens Gmbh H and Co- Bremen, D2800, Germany) at the speed of 25 rpm for 4 minutes, which should be in between 0.5–1% [16]. Following formula was used for the determination of percentage friability:


$$F=\;\frac{W_{o}-W_{t}}{W_{o}}*\text{1}\text{0}\text{0}$$ (6)

Where, W_o_ is the initial weight and W_t_ is the final weight of the tablets.

#### Fineness of dispersion test.

Fineness of dispersion test was performed by placing the 2 tablets from each formulation in 100 ml of water and shaken well and then the uniform dispersion was passed through the 710 μm mesh sieve [17].

#### Disintegration time.

Disintegration time was determined in accordance with USP by placing the tablets from each formulation in 100 ml beaker of water maintained at 15–25°C and the time was recorded. All tablets should be disintegrated within seconds [17].

#### Assay.

Assay was determined by making the 0.05% solution of tablets from each formulation in pH 6.8 buffer and diluted to 0.001%, similarly standard was prepared and then absorbance of both test and standard solutions was determined at 278 nm by using UV-Spectrophotometer (UV-1800 Shimadzu Corporation Kyoto, Japan). The % purity of Dimenhydrinate was found to be ≥ 98%. The % assay according to USP should be in between 90–110% [18].

#### Dissolution test.

For dissolution studies, USP paddle apparatus II was utilized containing 900 ml water as dissolution medium maintained at 37 ± 5°C. Apparatus was set at 50 rpm for 15 minutes [18].

### Thermal gravimetric analysis and differential scanning calorimetry (TGA-DSC)

API and selected formulation (F2) were analyzed by SDT 650 simultaneous TGA-DSC thermal analyzer. Approximately 4 mg of sample was placed in the sample holder. Also, a vacant aluminum reference holder was used as standard. Thermal assessment was done on the temperature range of 20–600°C, at the rate of 10°C/min. The flow rate was set at 99.98 mL/min in the active nitrogen atmosphere [19].

#### Scanning electron microscopy (SEM).

Scanning electron microscope (SEM) (JSM-6380A, Jeol, Japan) was used to analyze the morphological feature of optimized formulation (F2) at 15 kV. Results were observed at different magnifications in order to determine the particle size and interaction between the drug and excipients [19].

#### Compressional behavior analysis of optimized formulation.

Ten tablets from optimized formulation (F2) were compressed at different compressional pressure (13.789, 20.684, 27.579, 34.473 and 41.368 MN/m^2^) by using Dott Bonapace compression machine CPR 18 (Dott Bonapace & Co. Italy). At each compressional pressure (n = 3) tablets were compressed keeping constant mass of tablet and dimensions (within ± 1 mm). Manually the die cavity was filled each time for tablet compression after weighing the exact mass of powder blend. Compressed tablets were subjected to the Heckel analysis.

#### Heckle analysis.

Heckel equation explained the relationship between powder compression under the influence of applied pressure that follows first order reaction. When pressure is applied, the inter-particle space (i.e., porosity Ɛ) is decreased and hence densification of powder blend is achieved. Accordingly, a linear region of the curve (obtained from plotting [P] vs $\text{ln}\left[\frac{\text{1}}{\left(\text{1}-\rho_{\text{r}}\right)}\right]\;$ can be obtained as per given equation:


$$In\;\left[\frac{\text{1}}{\text{1}-pr}\right]=KP+A$$ (7)

Here $\rho_{𝐫}\;$ is the powder bed’s relative density and calculated as follows:


$$\rho_{𝐫}=\;\frac{\rho_{𝐀}}{\rho_{𝐓}}$$ (8)

The apparent density of tablet ($\rho_{𝐀}):$


$$\rho_{𝐀}=\;\frac{weight\;of\;tablet}{\pi r^{\text{2}}h}$$ (9)

(h = thickness of tablet and r = radius of tablet)

The true density $(\rho_{𝐓}$ of powder blend was calculated by reported procedure. The plot between applied pressure ‘P’ and ‘-ln Ɛ’ provides the value of slope (K) and intercept (A). With the help of value of K, mean yield pressure is obtained (1/K = P_Y_), which represents the material plasticity under compression. The smaller value of 1/K represents the higher plasticity of the material. The value of intercept ‘A’ provides useful information about densification of powder blend at different stages (D_O_ = densification of powder at die filling phase, D_A_ = densification of final compact and D_B_ = densification of powder during compression) [2].

#### Tensile strength measurement.

Tensile strength of compressed tablets was calculated with the help of following equation:


$$T=\frac{\text{2}F}{\pi DH\;}$$ (10)

Where, F is the crushing load (hardness), ‘H’ is the thickness in mm and ‘D’ is the diameter in mm of the tablets.

### *In vitro* release studies

For dissolution profile comparison, six tablets from each formulation were placed in paddle apparatus (USP apparatus II). F2 formulation was selected as a reference while remaining formulations were considered as test formulations. Different dissolution media (pH 1.2, pH 4.5, pH 6.8 and pH 7) were used, temperature was maintained at 37 ± 0.5°C at 50 rpm for 15 minutes [20]. Sampling was done at different time points, i.e., 2, 4, 6, 8, 10, 12 and 15 min. 10 ml sample was withdrawn and substituted at selected time intervals. Dilutions of sample and standard (0.001%) were prepared and their absorbance was taken at 278 nm with the help of UV Visible spectrophotometer.

### Methods to evaluate release kinetics

Different model dependent and independent methods were used for the assessment of drug release kinetics of F1-F9 formulations [21].

#### Model dependent methods.

***First- order kinetic model*:** This model is concentration dependent and explains the release of drug from the system. It is the time *vs.* log cumulative % drug remaining and is explained by following equation [21]:


$$\text{Log Q}=\text{Log}{\text{Q}}_{\text{0}}-\text{ kt}/\;\text{2}.\text{303}$$ (11)

Where, Q is the amount of drug release at time t, Q_o_ represents the initial concentration of drug and k is the first order rate constant.

***Higuchi kinetic model*:** It represents the time square root *vs.* cumulative % drug release [22]. This model is based on diffusion process and the Higuchi equation that describes the drug release is expressed as follows [23]:


$$Q=kt^{1/2}$$ (12)

***Hixson-Crowell kinetic model*:** It represents the time *vs*. cube root percentage drug remaining and can be described as follows [24]:


$${𝐐}_{0}^{1/3}-{𝐐}_{𝐭}^{1/3}={𝐊}_{HC}\times 𝐭$$ (13)

Where, Qo represents the initial concentration of the drug, Qt is the drug concentration at time t and K_HC_ represents the Hixson-Crowell rate constant [25].

***Weibull kinetic model*:** It represents the log of time *vs*. log of dissolved amount of drug [23]. The fraction of drug release in solution at time *t* is defined by Weibull model and its equation is expressed as follows:


$$𝐦=1-exp\;[\;{-(𝐭-Ti)}^{\beta }\;/\;\alpha ]$$ (14)

Or


Log [−ln (𝐥 −𝐦)]=𝐛 log (𝐭−Ti)−log α (15)

Where, T_i_ represents the lag time, α represents the time process and shape parameter is indicated by b. If b is equals to 1, it represents the exponential curve, less than 1 value of b indicates the parabolic with the elevated early slope and if it is greater than 1; it represents S-shaped with increasing curve followed by turning point [21].

The (*r*^2^ ≥ 0.99) for each model was calculated

#### Model independent methods.

In order to compare the drug release profiles of test and reference formulations, similarity factor (*f*_*2*_) and difference factor (*f*_*1*_) have been widely utilized and assessed by following equations [26]:


$$f_{1}=\;\left[\frac{\sum_{𝐭=1}^{𝐧}\left({𝐑}_{𝐭\;}–{𝐓}_{𝐭}\right)}{\sum_{𝐭=1}^{𝐧}{𝐑}_{𝐭}}\right]\times 100$$ (16)

Where, n is the number of samples, R_*t*_ is the % release of the reference formulation and T_t_ is the % release of test formulations.


$$f_{2}=50\times 𝐥𝐨𝐠\left\{{\left[{1+\left(\frac{1}{𝐍}\right)\sum \left(𝐑𝐢-𝐓𝐢\right)}^{2}\right]}^{-0.5}\right\}\times 100$$ (17)

Microsoft Excel ^TM^ 2013 and DD Solver^®^ program were used in order to analyze the data statistically and the best model was selected on the basis of highest value of *r*^2^ [27].

### Stability studies

International committee on harmonization (ICH) guidelines were followed for stability studies [14]**.** For the period of 6 months, all formulations were placed under accelerated conditions, i.e., 40°C ± 2°C and 75% ± 5% RH in humidity chamber. Samples were collected and observed at 0, 3 and 6 months for assay, disintegration and % drug release.

### *In silico* simulation of Dimenhydrinate by Physiologically Based Pharmacokinetic (PBPK) modelling

The present approach was to predict the plasma profile of reported plasma concentration *vs*. time profile of Immediate release (IR) dimenhydrinate 50 mg tablets from drug release data of optimized fast dispersible dimenhydrinate tablet (F2) using *in-silico* PBPK modeling based on the “Advanced Compartmental and Transit” (ACAT) model in software GastroPlus version 9.8 (SimulationsPlus Inc., Lancaster,CA, United States). The GastroPlus^TM^,has ADMET^TM^ predictor module which was used to estimate physico-chemical parameter of dimenhydrinate, i.e., effective jejuna permeability. The bioavailability of dimenhydrinate was reported by [28]. The values of molecular weight [29], Pka, elimination half life [28], log partition co efficient [30], plasma protein [31], fraction of the drug unbound in plasma, mean renal clearance [32], blood to plasma ratio [33] were from the reported literature [28–33] (S4 Table). For the validation purpose, fold error (FE) values (Eq 18) was used to establish predicted and observed parameters as follows:


$$FE=\frac{Predicted\;Value}{Observed\;Value}$$ (18)

## Result and discussion

Developing the fast dispersible tablets as patient friendly dosage form is an innovative approach to enhance the patient compliance by providing ease of administration, masking bitterness of API and improving bioavailability. This novel tableting technology is alternative to conventional dosage forms for aged patients, bedridden patients and pediatrics [34]. Intake of conventional tablets raise the issues of non-compliance, missed doses by the patients when dosing frequency increases. According to the survey, 30–40% elderly patients faced non-compliance and ineffectiveness of therapy due to dysphagia. Dispersible formulations disperse quickly as they contain super disintegrants that facilitates the quick disintegration and thus release the active drug rapidly, i.e., within seconds from the dosage form and ensure maximum bioavailability in minimum time [35]). In the present study nine different dimenhydrinate (50 mg) fast dispersible tablets were developed using avicel PH 102 and sodium starch glycolate as the independent variables at five different levels (Table 1), whereas hardness, disintegration time and % drug release at 15 mins were considered as response variables. Composition of dimenhydrinate fast dispersible formulations (F1-F9) were presented in Table 2.

### Pre formulation studies

Different tests were performed on powder blends to determine the flow behavior of powders and to develop uniform pharmaceutical product [34]. Bulk and tapped density were performed. All the formulations showed good flowability and compressibility behavior. The mean values of the Carr’s Index for F2 were 11.01 ± 0.65%. For hausner’s ratio and angle of repose the values for F2 were found to be 1.16 ± 0.04 and 33.32 ± 1.17º respectively (Table 3). Using powder flow characterization techniques, scientists assessed the powder blends of meloxicam using different tests [34]. Microcrystalline (MCC) PH 102 has a particle size of 100µm showing adequate flow behavior [36]. It deforms plastically which successfully increases the interparticles bonding area during compression. Thus compaction is improved due to the mechanical interlocking of irregular particles [2].

**Table 3:** Precompression Evaluation of Dimenhydrinate fast dispersible tablets.

Formulations	USP Comments	Carr’s Index	Hausner’s Ratio	Angle of repose	USP Comments
**F1**	Good	11.03 ± 0.60	1.13 ± 0.01	33.15 ± 0.71	Good
**F2**	Good	11.01 ± 0.65	1.16 ± 0.04	33.32 ± 1.17	Good
**F3**	Good	15.80 ± 0.53	1.18 ± 0.04	34.41 ± 1.26	Good
**F4**	Good	15.49 ± 0.56	1.13 ± 0.01	33.48 ± 1.26	Good
**F5**	Good	13.31 ± 0.99	1.16 ± 0.02	34.06 ± 0.76	Good
**F6**	Excellent	10.13 ± 0.74	1.11 ± 0.01	30.39 ± 0.23	Excellent
**F7**	Good	12.16 ± 0.73	1.18 ± 0.04	32.60 ± 0.56	Good
**F8**	Excellent	9.64 ± 0.52	1.09 ± 0.07	29.86 ± 0.54	Excellent
**F9**	Good	11.12 ± 0.79	1.18 ± 0.04	32 ± 0.67	Good

### Thermal gravimetric analysis/ differential scanning calorimetry

**Fig 1:**
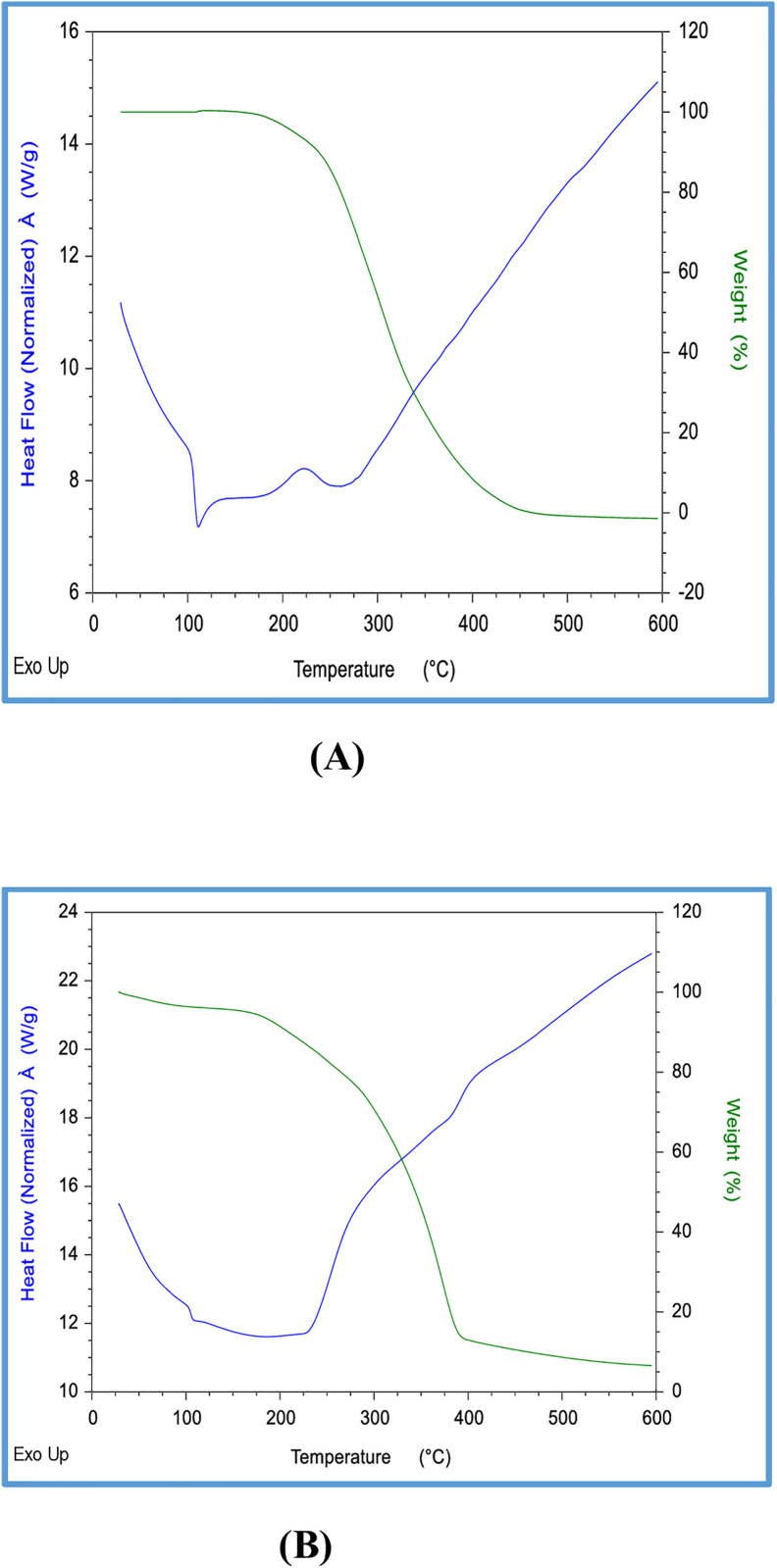
TGA/DSC of dimenhydrinate optimized formulation F2 (A), Dimenhydrinate (B).

The thermograms of F2 (formulation) and (API) revealed distinct melting points: approximately 235.79°C for F2 and 308.82°C for the API, accompanied by notable weight loss. The decomposition process, as indicated by the endothermic peaks on the heat flow curve, occurred within the temperature ranges of 90–270°C for the formulation and 28.81–227.65°C for the API, suggesting initial crystallinity of the materials. Notably, decomposition of the samples was anticipated at temperatures exceeding 375°C for the optimized formulation and 300°C for the API, as demonstrated in (Fig 1A and 1B).

### Scanning electron microscopy

**Fig 2:**
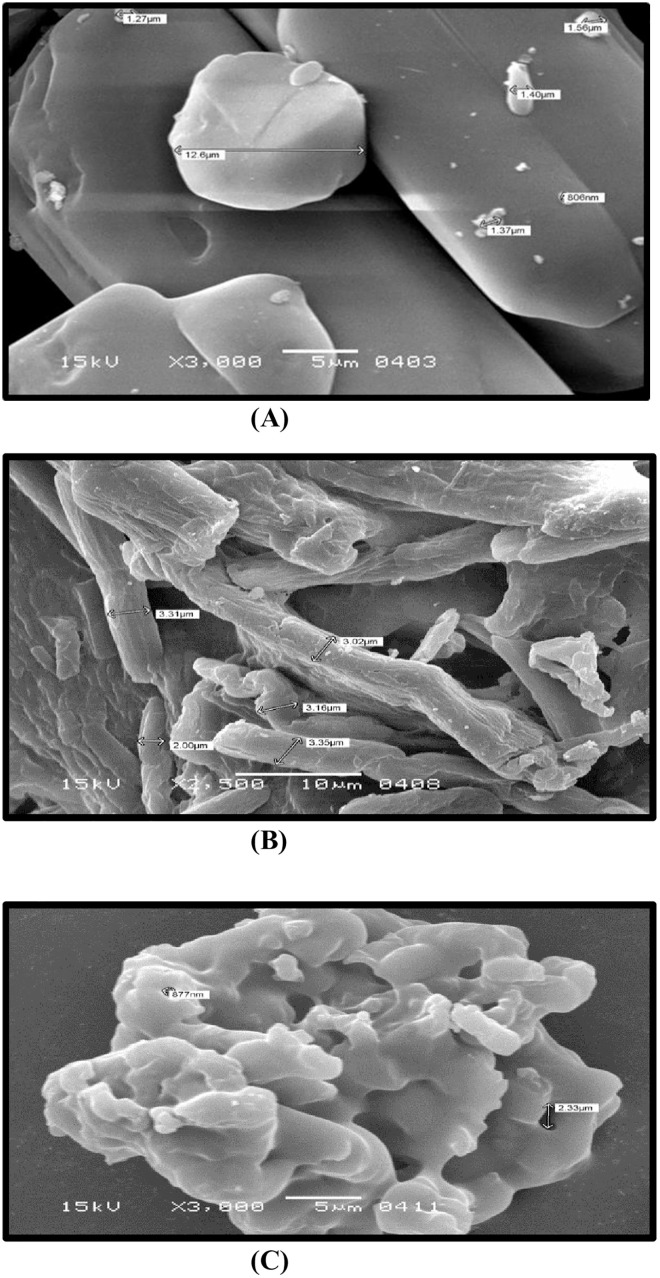
Scanning electron microscopy (SEM) of Dimenhydrinate (A), dimenhydrinate with Avicel PH102 (B) and dimenhydrinate with sodium starch glycolate (C) at 15 kv.

Scanning electron microscope (SEM) studies were also performed. Morphological features like shape, size, surface attributes, particle size and aggregation can be outsized by scanning electron microscopy that uses a beam of electron for visual investigation. To achieve the highest resolution of particles, SEM images are captured at various magnifications. Analysis of pure dimenhydrinate (Fig 2A) revealed particles shaped like rods, varying in size and measured in micrometers. When combined with avicel PH 102 (Fig 2B) and sodium starch glycolate (Fig 2C). SEM studies depicted a clustered or agglomerated structure with rod-shaped particles possessing smooth surfaces.

### Physicochemical evaluation studies

**Fig 3:**
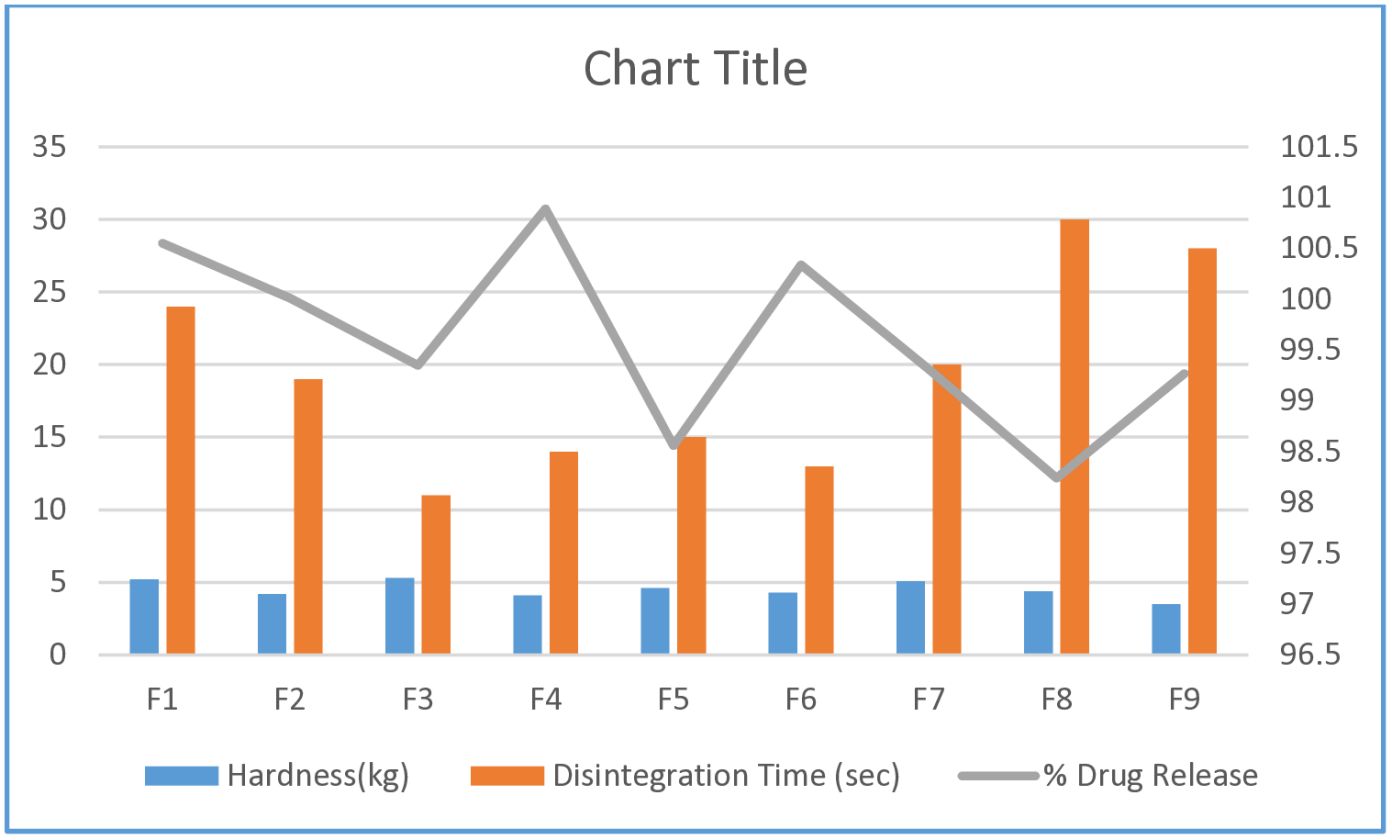
Graphical representation of hardness, disintegration time and % drug release of F1-F9.

In the present study, different pharmaceutical quality assessments were carried out. Results of weight variation test of F1-F9 were found to be 154.20 ± 4.99 to 158.64 ± 3.68 mg. The mean thickness and diameter of different formulations were found to be 2.506 ± 0.01 to 2.64 ± 0.17 mm and 8.42 ± 0.08 to 8.49 ± 0.04 mm respectively. Similarly, % friability of F1-F9 were ranged of 0.60 to 0.83%. Tablets showed no crack, split or cracking signs after the tests. F1- F9 also passes the fineness of dispersion test. The % assay were found to be 90.56 ± 0.57% to 100.18 ± 0.55%, as per USP standards (90–110%) (Table 4). The hardness, disintegration time and single point dissolution test of F1-F9 were graphically represented in Fig 3. Formulation F2 was found to be the optimized formulation with good mechanical strength, i.e., hardness: 4.2 kg, friability: 0.77%, disintegration time 19 secs and % drug release 100.01% at 15 minutes (Table 4 and Fig 3).The research showed that an increase in the concentration of avicel (microcrystalline cellulose) used as a filler or binder leads to a rise in the hardness of the tablet. To enhance the mechanical strength of tablets produced through direct compression, several excipients are available in the local market. These materials offer suitable compactibility and flowability, demonstrating an excellent dissolution rate necessary for creating tablets with an immediate release profile [37]. Scientists reported that the disintegration time and hardness presented noteworthy relationship in the development of fast-dispersible paracetamol tablets [38].Disintegration and *in vitro* drug release of tablet are mainly dependent on its porosity, friability, hardness, while friability of such formulations should be less than 1% [39].In the present study, use of sodium starch glycolate (2–8%) yielded the disintegration time in the range of 13–30 secs, among which the optimized formulation F2 showed disintegration time of 19 secs. This ingredient has been successfully used in many researches for producing fast disintegration by swelling mechanism [40] F1-F9 successfully passed the dispersion finesse test, and the assay test observations were within the acceptable range (Table 4, Fig 3).

**Table 4:** Quality attributes of Dimenhydrinate fast dispersible tablet formulations.

PARAMETERS	F1	F2	F3	F4	F5	F6	F7	F8	F9
**Weight Variation (mg)**	156.42 5.53±	155.92 5.54±	154.20 4.99±	156.77 5.15±	157.22 4.10±	156.22 4.13±	157.22 4.10±	156.65 3.68±	158.64 3.68±
**Thickness (mm)**	2.506 ± 0.01	2.566 ± 0.08	2.51 ± 0.06	2.558 ± 0.07	2.559 ± 0.06	2.527 ± 0.03	2.614 ± 0.17	2.531 ± 0.06	2.561 ± 0.15
**Diameter (mm)**	8.42 ± 0.13	8.46 ± 0.06	8.45 ± 0.08	8.47 ± 0.07	8.45 ± 0.06	8.42 ± 0.08	8.42 ± 0.08	8.49 ± 0.04	8.46 ± 0.05
**Friability**	0.81	0.77	0.73	0.83	0.72	0.66	0.82	0.60	0.74
**Finess of dispersion**	All formulations dispersion passes from 710μm mesh sieve								
**Assay (%)**	90.89 ± 0.67	96.30 ± 0.95	90.56 ± 0.57	90.63 ± 0.70	98.08 ± 0.54	90.63 ± 0.71	99.86 ± 0.54	100.18 ± 0.55	99.67 ± 0.77

### Single point dissolution

*In-vitro* tests are performed to assess the quality standard of pharmaceutical dosage forms. These tests are highly recommended by the regulatory authorities in order to depict the *in-vivo* assessment. *In vitro* data can be generated by various dissolution tests. The absorption of BCS class II and IV drugs can easily be assessed with the help of these dissolution studies [41]. In the present study, results of single point dissolution were found in acceptable limits (Fig 3).

**Fig 4:**
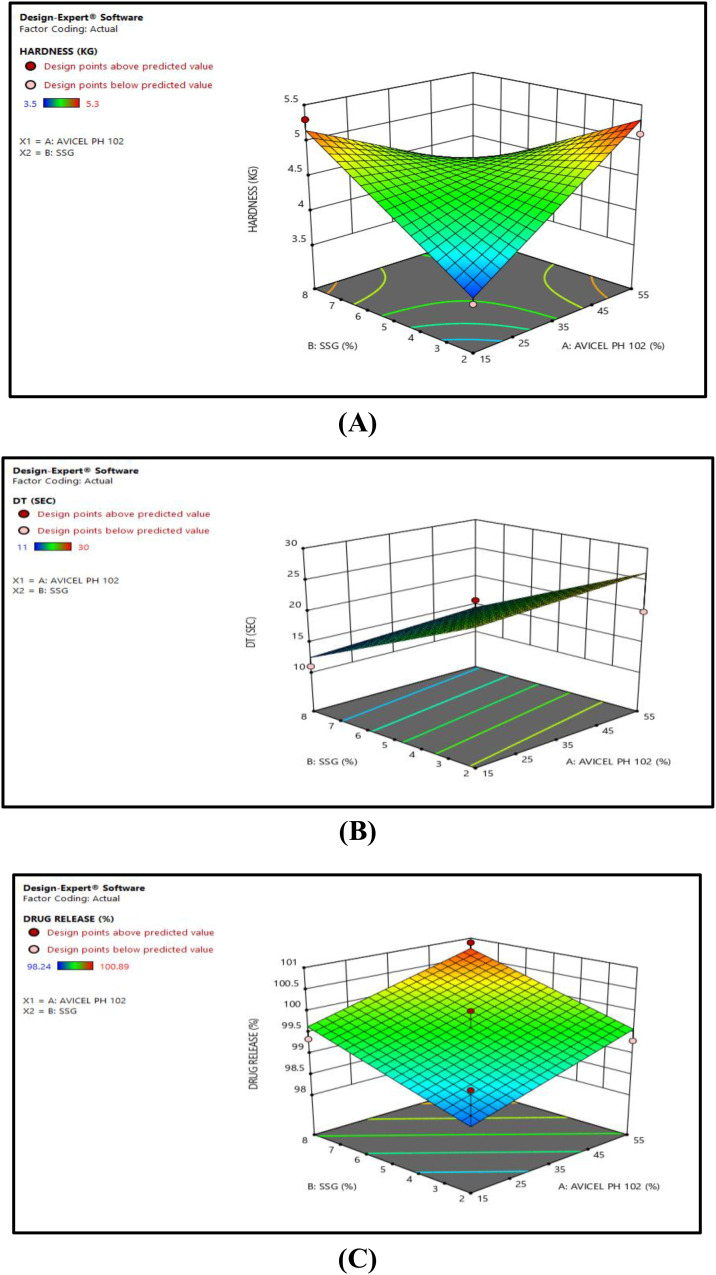
3D response surface plots presenting the effects of independent variables on Hardness (A), (B) and % drug release (C) of F1 – F9.

**Formulation optimization for Dimenhydrinate Fast dispersible tablets:** Central composite design is considered as the most preferable method as compared to the other optimization methods. It provides contour plots and polynomial equations as well as statistical model summary to understand the significance of model [42]. In the present study, design model was found to be Quadratic. The impact of two independent variables, i.e., avicel PH 102 (X_1_) and sodium starch glycolate (X_2_) were determined on three dependent variables (hardness test, disintegration time and % drug release at 15 min). RSM plot for hardness test (R_1_) of F1 – F9 was illustrated in Fig 4(A) which indicated that as the concentration of avicel PH 102 increases, the hardness of the dispersible tablets were also improved. The ANOVA summary of hardness tests represents the F-value was 6.28 and *p* value was 0.037, indicating that the 2FI model was valid and significant. The Adeq Precision for hardness test was found to be 7.498 represents an acceptable signal-to-noise ratio. Whereas the values of R^2^, adjusted R^2^ and predicted R^2^ were recorded as 0.790, 0.664 and 0.425 respectively (Tables 5, 6 and S1). RSM plot of effects of excipients on disintegration time (R_2_) was demonstrated in Fig4(B) that an increase in the concentration of sodium starch glycote decreases the disintegration time of the tablets. The optimum concentration of binder and super disintegrant should be used to obtain the tensile strength of the tablets and to obtain faster disintegration of the tablet. For disintegration test, F-value and *p*-value were found to be 10.16 and 0.011 respectively, indicating that Linear model was significant with acceptable signal to noise ratio. The values of Adequate precision, R^2^, adjusted R^2^and predicted R^2^ were found to be 7.702, 0.772, 0.695 and 0.441 respectively (Tables 5, 6 and S2)). The RSM plot of effects of excipients on % drug release (R_3_) at 15 minutes, showed in Fig 4(C) which explained the impact of both the excipients (Avicel PH 102 (X_1_) and sodium starch glycol ate (X_2_) on % drug release at 15 minutes. The F-value and *p*-value were found to be 11.12 and 0.009 respectively demonstrated that Linear model were significant with admissible signal to noise ratio. For % drug release at 15 minutes, the values of Adequate precision, R^2^, adjusted R^2^, and predicted R^2^ were recorded as 8.164, 0.787, 0.716 and 0.508 respectively (Tables 5, 6 and S3). The *p* value, F-value and the final equations in terms of actual factors for R_1_, R_2_ and R_3_ were expressed in (Table 5).

**Table 5:** Probability value, F- value and actual equations of selected responses.

Responses	*p* value	F- value
R_1_ (Hardness)	0.037	6.28
R_2_ (Disintegration)	0.011	10.16
R_3_ (% drug release at 15 mins)	0.009	11.12
**Actual Equations for Responses**		
Y1R_1_= +4.52+0.1561*A+0.0823*B−0.7000*AB		
Y2 R_2_= +19.33+0.9660*A−5.88*B		
R_3_ = +99.61+0.5493*A+0.5787*B		

**Table 6:** Fit summary statistics for hardness, disintegration time and % drug release at 15 min of F1-F9.

Response	Source	Sequential *p-value*	R^2^	Adjusted R²	Predicted R²	Adeq. Precision	Remarks
Hardness (R_1_)	2FI	0.009	0.790	0.664	0.425	7.498	Suggested
Disintegration time (R_2_)	Linear	0.011	0.772	0.695	0.441	7.702	Suggested
% Drug Release at 15 min (R_3_)	Linear	0.009	0.787	0.716	0.508	8.164	Suggested

### *In vitro* drug release studies

#### Model dependent method.

**Fig 5:**
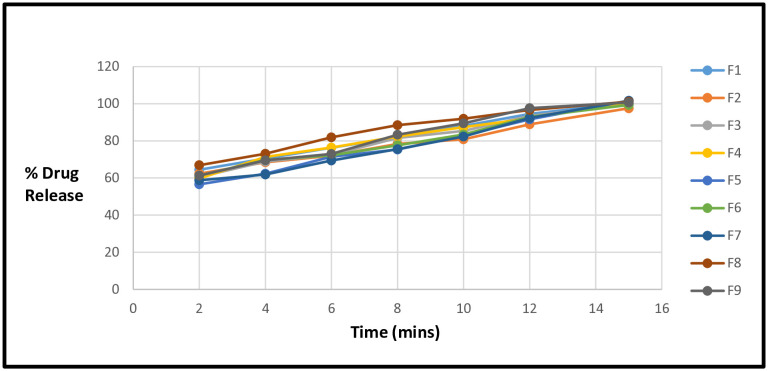
Graphical Representation of % Drug release (F1-F9) at pH 7.

The dissolution rate of a drug substance transitioning from a solid to a solution state per unit time is assessed through *in vitro* drug release studies conducted under specific conditions. Additionally, dissolution testing can also help ascertain the physicochemical stability of a drug product. The choice of method for analyzing the release rate and quantity of the drug in the sample is crucial and requires careful consideration. UV-spectrophotometry is the most commonly employed technique for the quantitative analysis of the active pharmaceutical ingredient (API) in *in vitro* studies [43]. In this study, release profiles were compared at four different dissolution media. Fig 5 presented the release profile of F1-F9 at pH 7. Results were analyzed using different kinetic models to determine the release behavior of these formulations (Table 7). Scientists assessed *in vitro* drug release profiles using three dissolution media (0.1N HCl, phosphate buffer at pH 4.5, and pH 6.8). They employed first-order, zero-order, and hixson-crowell models to analyze the kinetic behavior of the drug release [20,21]. In this study, the *r*^2^ values for first-order kinetic model, higuchi, hixon crowell and weibull model for F2 at pH 1.2 were 0.404, 0.934, 0.246 and 0.974 respectively (Table 7). For F2 at pH 4.5, the *r*^2^ values were 0.47, 0.939, 0.292 and 0.983 for first-order kinetic model, higuchi, hixon crowell and weibull model respectively (Table 7). For F2 at pH 6.8 and pH 7, the *r*^2^ values were 0.701 and 0.465, 0.916 and 0.075, 0.526 and 0.042, 0.937 and 0.883 for first-order kinetic model, higuchi, hixon crowell and weibull model respectively (Table 7). The findings in the present study revealed that all formulations followed to the weibull model, with the excellent *r*^*2*^ values observed at pH 6.8, ranged from 0.929–0.962 (Table 7). DD-solver software was used for the determination of coefficient of regression (*r*^*2*^) for all kinetics models. According to the results, all formulations followed Weibull model. Scienists reported the comparison of different brands of mefenamic acid tablets. Results indicated that all brands followed Weibull kinetic model [39].

**Table 7:** Release kinetics of Dimenhydrinate fast dispersible tablets at Different Dssolution media.

Formulations	First Order		Higuchi		Hixon Crowell		Weibull Model		
	*r* ^2^	*k1* ^*(h-1)*^	*r* ^*2*^	kH^(h-1/2)^	*r* ^*2*^	kHC^(h-1/3)^	*r* ^*2*^	α	Β
**pH 1.2**									
F1	0.207	0.060	0.884	13.904	0.003	0.018	0.974	5.132	0.469
F2	0.404	0.058	0.934	13.618	0.246	0.017	0.974	5.709	0.505
F3	0.240	0.059	0.890	13.814	0.047	0.018	0.969	5.238	0.475
F4	0.317	0.055	0.913	13.185	0.158	0.017	0.959	5.733	0.488
F5	0.291	0.056	0.914	13.228	0.125	0.017	0.967	5.643	0.482
F6	0.260	0.058	0.905	13.607	0.074	0.017	0.977	5.378	0.478
F7	0.375	0.058	0.918	13.587	0.219	0.017	0.958	5.661	0.500
F8	0.279	0.060	0.901	13.951	0.087	0.018	0.975	5.248	0.482
F9	0.302	0.059	0.910	13.826	0.117	0.018	0.978	5.357	0.486
**pH 4.5**									
F1	0.356	0.074	0.898	16.032	0.138	0.022	0.979	4.493	0.499
F2	0.471	0.071	0.939	15.548	0.292	0.021	0.983	4.945	0.523
F3	0.459	0.073	0.927	15.837	0.265	0.021	0.985	4.792	0.521
F4	0.289	0.072	0.875	15.745	0.068	0.021	0.965	4.487	0.486
F5	0.372	0.074	0.904	15.982	0.154	0.021	0.985	4.542	0.502
F6	0.472	0.071	0.929	15.607	0.288	0.021	0.979	4.928	0.524
F7	0.339	0.073	0.885	15.823	0.122	0.021	0.969	4.550	0.496
F8	0.261	0.071	0.876	15.592	0.046	0.021	0.962	4.498	0.481
F9	0.480	0.071	0.931	15.581	0.298	0.021	0.979	4.962	0.526
**pH 6.8**									
F1	0.781	0.128	0.954	21.816	0.637	0.035	0.954	3.843	0.654
F2	0.701	0.132	0.916	22.075	0.526	0.036	0.937	3.478	0.617
F3	0.781	0.137	0.944	22.460	0.635	0.037	0.946	3.674	0.659
F4	0.837	0.120	0.979	21.150	0.724	0.033	0.962	4.381	0.689
F5	0.677	0.122	0.922	21.248	0.496	0.033	0.946	3.613	0.603
F6	0.716	0.115	0.939	20.737	0.575	0.032	0.929	3.972	0.626
F7	0.734	0.126	0.940	21.573	0.574	0.034	0.947	3.723	0.630
F8	0.803	0.142	0.947	22.890	0.662	0.038	0.949	3.647	0.673
F9	0.835	0.128	0.973	21.837	0.717	0.035	0.958	4.149	0.691
**pH 7**									
F1	0.441	0.313	0.083	28.872	0.011	0.082	0.873	1.582	0.537
F2	0.465	0.313	0.075	28.884	0.042	0.081	0.883	1.593	0.542
F3	0.468	0.321	0.085	29.065	0.053	0.084	0.868	1.575	0.545
F4	0.450	0.319	0.081	29.015	0.029	0.083	0.864	1.572	0.541
F5	0.508	0.315	0.236	29.117	0.143	0.082	0.848	1.635	0.564
F6	0.495	0.314	0.179	29.019	0.110	0.081	0.860	1.622	0.556
F7	0.429	0.329	0.017	29.186	0.002	0.086	0.854	1.529	0.536
F8	0.453	0.333	0.017	29.291	0.036	0.086	0.855	1.529	0.542
F9	0.493	0.327	0.108	29.269	0.106	0.084	0.856	1.577	0.556

#### Model independent methods.

To evaluate *in vitro* data, test formulations (F1 - F7 and F9) were assessed using the reference formulation (F2) following similarity factor (*f*_*2*_) and difference factor (*f*_*1*_). The model-independent approach is primarily employed for three purposes: introducing new products, adjusting existing compound formulations, and comparing formulations with those available in the market. The analysis of the *f*_*1*_ and *f*_*2*_ values indicated a similarity in profiles across various pH conditions [25]. Outcomes of model independent method were within acceptable limits. Results indicated that the release pattern of test formulations were shown as alike with the F2 (reference formulation). At pH 1.2, 4.5, 6.8 and 7, *f*_*1*_ values were ranged from 0.73–5.63, 1.55–3.36, 1.85–9.47 and 0.55–1.50 respectively and *f*_*2*_ values ranged from 78.06–98.75, 85.93–92.46, 59.54–88.95 and 88.13–96.60 respectively (Table 8).

**Table 8:** Similarity and difference factors for Dimenhydrinate fast dispersible test formulations.

Formulations	*f*_1_	*f*_2_
**PH 1.2**		
**F1 & F8**	0.73	98.75
**F2 & F8**	2.68	90.88
**F3 & F8**	1.11	97.42
**F4 & F8**	5.63	78.06
**F5 & F8**	5.27	78.84
**F6 & F8**	2.46	90.05
**F7 & F8**	2.95	87.66
**F9 & F8**	1.39	96.69
**PH 4.5**		
**F1 & F8**	2.97	86.81
**F2 & F8**	1.55	92.32
**F3 & F8**	3.03	86.92
**F4 & F8**	1.76	92.46
**F5 & F8**	3.36	85.93
**F6 & F8**	2.96	88.23
**F7 & F8**	3.10	86.83
**F9 & F8**	2.94	87.22
**PH 6.8**		
**F1 & F8**	4.76	74.30
**F2 & F8**	3.57	78.56
**F3 & F8**	1.85	88.95
**F4 & F8**	8.08	63.67
**F5 & F8**	6.91	65.38
**F6 & F8**	9.47	59.54
**F7 & F8**	5.67	69.54
**F9 & F8**	4.97	72.99
**PH 7**		
**F1 & F8**	1.50	88.13
**F2 & F8**	1.45	89.00
**F3 & F8**	0.84	94.44
**F4 & F8**	1.01	92.95
**F5 & F8**	1.07	91.60
**F6 & F8**	1.23	90.40
**F7 & F8**	0.55	96.60
**F9 & F8**	0.82	95.50

### Compressional behavior analysis

Evaluating the compressibility of powder blend prior compression is one of the dominant material properties for explaining the behavior of mixture exposed to the die cavity for tableting. Compressibility of powder is the removal of inter-particle space under influence of applied pressure [44]. Each ingredient in a powder blend possess its own characteristics which should be explored before compressing into a unit dosage form to ensure stable and smooth manufacturing process; for yielding the tablets having sufficient mechanical strength [45]. Many researchers have proposed methods to determine compressibility, amongst them Heckel equation is most widely employed.

In this study for executing the Heckel analysis the compacts were compressed from the optimized formulation at exact weight and dimensions (within ±1 mm) whereas the true density was calculated (1.55 gm/cm^3^) as per reported procedure using various compressional pressures: 13.789, 20.684, 27.684, 34.473, and 41.368 MN/m^2^. The mean data was used to establish the Heckel plot (i.e., compressional pressure [P] *vs* $\text{ln}\left[\frac{\text{1}}{\left(\text{1}-\rho_{\text{r}}\right)}\right]\;$.

**Fig 6:**
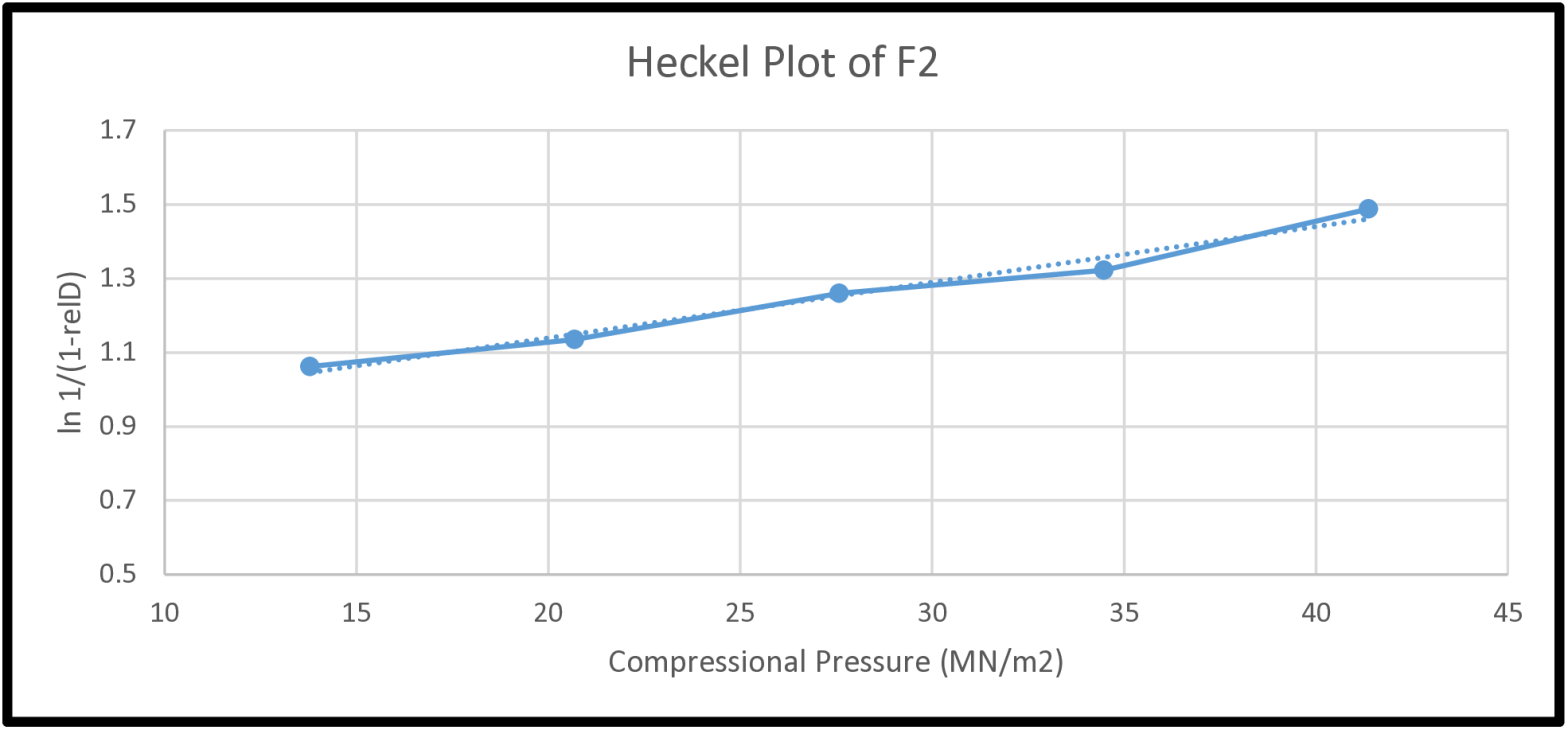
Heckel plot of optimized formulation (F2) at different compressional pressure.

The value of P_Y_ (yield value) of the optimized formulation was found to be 66.666 MN/m^2^ (Table 9 and, Fig 6). It has been reported that a low P_Y_ value exhibits the maximum plasticity of powder blend [2]. With exact specification of the testing conditions and reported P_y_ values of selected excipients it is possible to draw a conclusion based on comparative analysis to predict powder compressibility behavior. For characterization of new substance or powder blend with unknown deformation pattern, Heckel analysis is viable even in the absence of reported values. Although there are much criticized reports available on Heckel analysis (In die/out of die analysis of compact) but still it is the most discussed method of compressional behavior determination [44].

**Table 9:** Parameters of Heckle Equation of optimized formulation (F2).

HECKEL EQUATION PARAMETERS			
D_0_	D_A_	D_B_	P_Y_ (MN/m^2^)
0.711	0.081	0.369	66.666

### Tensile strength and hardness

Tensile strength and hardness serve as indicators of mechanical strength, reflecting the bonding between particles. In the context of tablet dosage forms, it is advisable to assess the mechanical properties of the primary ingredient to anticipate the overall compression behavior of the tablets [2]. The mechanical properties were evaluated by measuring tensile strength, which ranged from 1.093 ± 1.66 to 1.642 ± 1.76 MN/m^2^ (Table 10). Results suggested robust inter-particle bonding of powder blends. Additionally, the % friability of the tablets ranged from 0.60–0.83% (Table 4).

**Table 10:** Tensile strength of the optimized formulation (F2) at different compressional pressure.

COMPACTION PRESSURE (MN/m^2^)	TENSILE STRENGTH (MN/m^2^)
**13.789**	1.093 ± 1.66
**20.684**	1.112 ± 2.32
**27.579**	1.211 ± 2.34
**34.473**	1.520 ± 3.21
**41.368**	1.642 ± 1.76

### Stability testing

**Fig 7:**
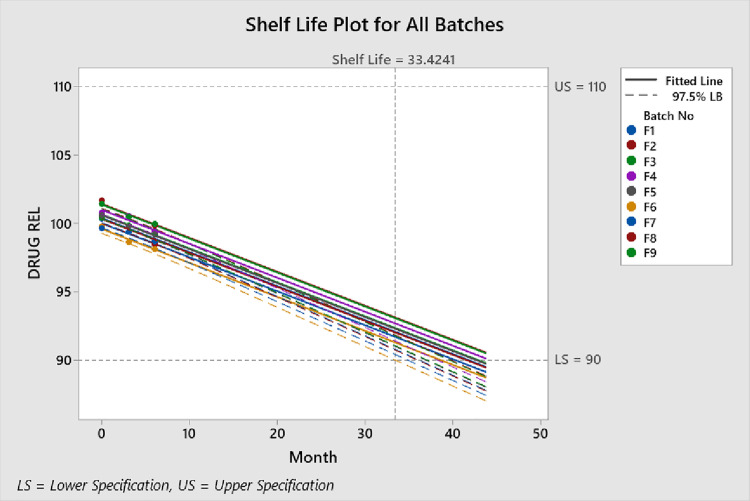
Shelf-life plots of Dimenhydrinate fast dispersible formulations (F1-F9) kept at accelerated stability testing.

All nine formulations demonstrated excellent quality features, i.e., assay, disintegration time and % drug release studies (Table 11). Stability studies of pharmaceutical dosage form allows estimation of shelf life of the products through application of statistical software [46]. Minitab was employed to estimate the shelf life of optimized formulation kept under accelerated storage conditions for stability testing. The shelf life of all formulations were presented in Fig 7.

**Table 11:** Stability studies of Dimenhydrinate fast dispersible tablets for 0 to 6 months at accelerated conditions.

FORMULATIONS	PARAMETERS								
	0 MONTH			3 MONTHS			6 MONTHS		
	Assay (%) n = 3	D.T (secs) n = 6	Dissolution Test (%) n = 3	Assay (%) n = 3	D.T (secs) n = 6	Dissolution Test (%) n = 3	Assay (%) n = 3	D.T (secs) n = 6	Dissolution Test (%) n = 3
**F1**	90.89 ± 0.67	13	96.88 ± 0.008	90.53 ± 0.45	15	96.55 ± 0.004	89.94 ± 0.22	15	95.55 ± 0.002
**F2**	96.30 ± 0.95	21	99.31 ± 0.008	96.12 ± 0.88	21	99.12 ± 0.004	94.12 ± 0.45	20	98.12 ± 0.004
**F3**	90.56 ± 0.57	16	98.55 ± 0.01	90.24 ± 0.45	17	97.34 ± 0.02	89.45 ± 0.34	17	96.23 ± 0.03
**F4**	90.63 ± 0.70	15	96.35 ± 0.007	90.12 ± 0.54	17	96.12 ± 0.003	90.03 ± 0.23	17	96.10 ± 0.003
**F5**	98.08 ± 0.54	18	98.33 ± 0.01	97.12 ± 0.34	18	97.24 ± 0.01	96.22 ± 0.24	19	96.11 ± 0.02
**F6**	90.63 ± 0.71	19	97.57 ± 0.01	90.34 ± 0.32	19	95.12 ± 0.02	90.12 ± 0.35	20	94.24 ± 0.04
**F7**	99.86 ± 0.54	16	99.55 ± 0.02	99.45 ± 0.22	17	98.45 ± 0.02	98.23 ± 0.24	17	96.34 ± 0.03
**F8**	100.18 ± 0.55	19	96.45 ± 0.01	100.15 ± 0.45	19	96.55 ± 0.01	99.67 ± 0.34	18	97.34 ± 0.02
**F9**	99.67 ± 0.77	17	95.33 ± 0.01	98.45 ± 0.45	18	94.32 ± 0.01	97.23 ± 0.34	18	94.12 ± 0.02

### Physiologically based pharmacokinetic modeling

**Fig 8:**
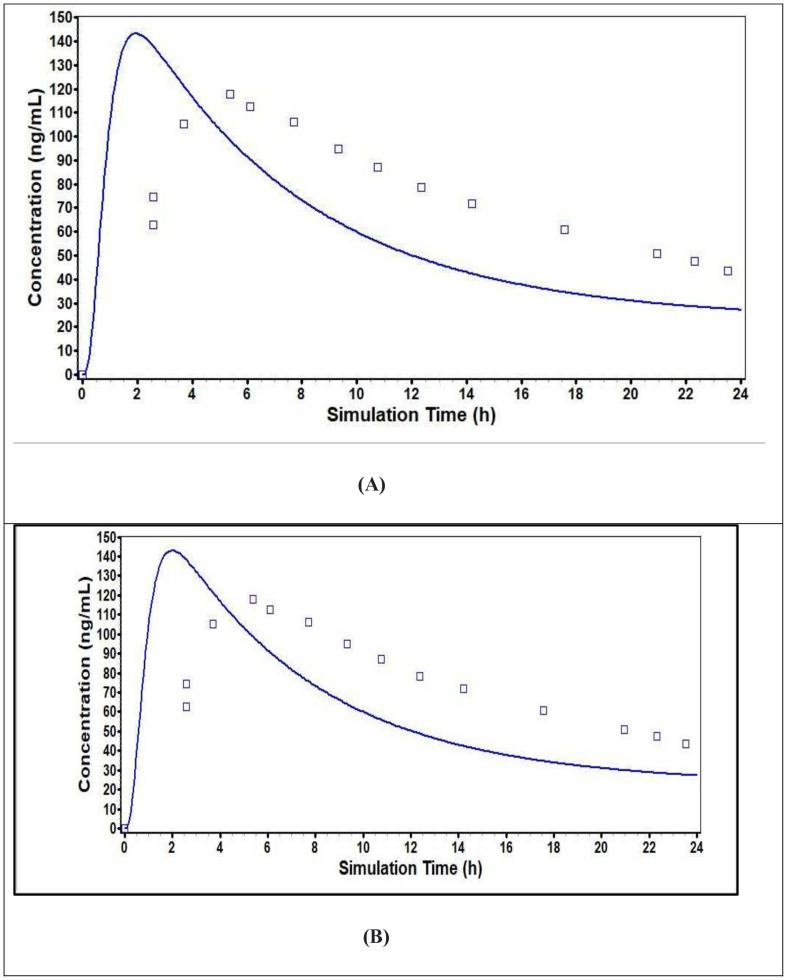
Predicted and observed Plasma Concentration *vs*. Time Profile of IR Dimenhydrinate 50 mg tablet (A) with F2 (optimized formulation) (B).

The prediction about the systemic disposition for the optimized Dimenhydrinate fast dispersible tablets (F2) was carried out using the TCAT® model. The pharmacokinetic parametric values and plasma profiles for dimenhydrinate immediate release (IR) tablets collected from reported literature (S4 Table File) against the predicted plasma profiles of (F2) optimized formulation are shown in Table 12 and Fig 8. The mean pharmacokinetic and simulated values for dimenhydrinate immediate release (IR) tablets were found to be 117.93 ng/ml and 143.3 ng/ml (C_*max*_), 5.3779 h and 1.92 h (T_*max*_), 2343.1 ng-h/ml and 2534.2 ng-h/ml (AUC _0-inf_) and 1732.5 ng-h/ml and 1477.9 ng-h/ml (AUC _0-t_). Simulated PK values of F2 were found to be 143.16 ng/ml (C_*max*_), 2 h (T_*max*_), 2533.8 ng-h/ml (AUC _0-inf_) and 1477.9 ng-h/ml (AUC _0-t_) as illustrated in Table 12. Results indicated that the fold error value was > 2 which indicated that experimental values are comparable with the predicted values.

**Table 12:** Reported [28-33} and predicted values of Dimenhydrinate pharmacokinetic parameters with Fold Error (FE).

Pharmacokinetic Parameters	Dimenhydrinate 50 mg Immediate Release (IR) Tablets			Optimized Fast Dispersible Dimenhydrinate Tablet (F2)		
	Observed	Predicted	Fold error	Observed	Predicted	Fold Error (FE)
C_*max*_ ng/ml	117.93	143.3	1.215	117.93	143.16	1.215
T_*max*_	5.3779	1.92	0.357	5.3779	2	0.357
AUC_*0-inf* (_ng-h/ml)	2343.1	2534.2	1.081	2343.1	2533.8	1.081
AUC _*0-t*_ (ng-h/ml)	1732.5	1477.9	0.853	1732.5	1477.9	0.853

## Conclusion

In this study, fast dispersible Dimenhydrinate tablets were developed and the impact of avicel PH102 and sodium starch glycolate were calculated. Heckel equations were used to determine the compressional behavior of the optimized formualtion. Formulation (F2) showed excellent compactional strength with quick disintegration time and excellent % drug release. The PBPK model is applied satisfactorily to estimate the plasma drug concentration *vs.* time. Thus by selecting appropriate concentrations of excipients a stable, elegant dosage form can be devloped for the patient.

## Supporting information

S1 Table Summary of ANOVA results for Hardness. (DOCX)

S2 Table Summary of ANOVA results for Disintegration Time. (DOCX)

S3 Table Summary of ANOVA results for % drug release at 15 min. (DOCX)

S4 Table Physiological and physicochemical dimenhydrinate parameters. (DOCX)
