# Supplementary material for: Development and characterisation of fast dispersible dimenhydrinate tablets: Compactional study and in-silico PBPK modeling
Source: PLoS One. 2025 Oct 27;20(10):e0334421. doi: 10.1371/journal.pone.0334421 (PMC12558512; doi:10.1371/journal.pone.0334421)
Supplement: S1 Table — (DOCX) [file pone.0334421.s001.docx]

**Table S1: Summary of ANOVA results for Hardness**

| **Source** | **Sum of Squares** | **Df** | **Mean Square** | **F-value** | **p-value** | **Remarks** |
| --- | --- | --- | --- | --- | --- | --- |
| **Model** | 3.45 | 3 | 1.15 | 8.28 | 0.0220 | significant |
| A-MCC | 0.4697 | 1 | 0.4697 | 3.38 | 0.1255 |  |
| B-SSG | 1.66 | 1 | 1.66 | 11.94 | 0.0181 |  |
| AB | 1.32 | 1 | 1.32 | 9.51 | 0.0274 |  |
| **Residual** | 0.6954 | 5 | 0.1391 |  |  |  |
| **Cor Total** | 4.15 | 8 |  |  |  |  |
