# Supplementary material for: Development and characterisation of fast dispersible dimenhydrinate tablets: Compactional study and in-silico PBPK modeling
Source: PLoS One. 2025 Oct 27;20(10):e0334421. doi: 10.1371/journal.pone.0334421 (PMC12558512; doi:10.1371/journal.pone.0334421)
Supplement: S2 Table — (DOCX) [file pone.0334421.s002.docx]

**Table S2: Summary of ANOVA results for Disintegration Time**

| **Source** | **Sum of Squares** | **Df** | **Mean Square** | **F-value** | **p-value** | **Remarks** |
| --- | --- | --- | --- | --- | --- | --- |
| **Model** | 332.49 | 2 | 166.24 | 26.43 | 0.0011 | Significant |
| A-MCC | 41.92 | 1 | 41.92 | 6.67 | 0.0417 |  |
| B-SSG | 290.56 | 1 | 290.56 | 46.20 | 0.0005 |  |
| **Residual** | 37.73 | 6 | 6.29 |  |  |  |
| **Cor Total** | 370.22 | 8 |  |  |  |  |
