# Supplementary material for: Development and characterisation of fast dispersible dimenhydrinate tablets: Compactional study and in-silico PBPK modeling
Source: PLoS One. 2025 Oct 27;20(10):e0334421. doi: 10.1371/journal.pone.0334421 (PMC12558512; doi:10.1371/journal.pone.0334421)
Supplement: S3 Table — (DOCX) [file pone.0334421.s003.docx]

**Table S3: Summary of ANOVA results for % Drug Release at 15 min**

| **Source** | **Sum of Squares** | **Df** | **Mean Square** | **F-value** | **p-value** | **Remarks** |
| --- | --- | --- | --- | --- | --- | --- |
| **Model** | 3.41 | 3 | 1.14 | 5.54 | 0.0479 | significant |
| A-MCC | 0.0363 | 1 | 0.0363 | 0.1769 | 0.6915 |  |
| B-SSG | 0.0280 | 1 | 0.0280 | 0.1363 | 0.7271 |  |
| AB | 3.35 | 1 | 3.35 | 16.30 | 0.0099 |  |
| **Residual** | 1.03 | 5 | 0.2054 |  |  |  |
| **Cor Total** | 4.44 | 8 |  |  |  |  |
