# Supplementary material for: Development and characterisation of fast dispersible dimenhydrinate tablets: Compactional study and in-silico PBPK modeling
Source: PLoS One. 2025 Oct 27;20(10):e0334421. doi: 10.1371/journal.pone.0334421 (PMC12558512; doi:10.1371/journal.pone.0334421)
Supplement: S4 Table — (DOCX) [file pone.0334421.s004.docx]

**Table S4: Physiological and Physicochemical Dimenhydrinate Parameters**

| **Parameters** | **Values** | **References** | |
| --- | --- | --- | --- |
| Bioavailability | 43-72% | (Abdul Rasool et al., 2021) [28] | |
| Molecular weight | 469.96 | (Rivai et al., 2018) [29] | |
| Pka | 8.87 | (Abdul Rasool et al., 2021) [28] | |
| Elimination Half life | 1-4 hours | (Abdul Rasool et al., 2021) [28] | |
| Log partition  co efficient | 0.63 | (Leichner et al., 2019) [30] | |
| Plasma protein | 98% | (Sicari et al., 2025) [31] | |
| Fraction of the drug unbound in plasma | 0.216 | (Adachi et al., 2021) [32] | |
| Mean Renal clearance (L/h) | 0.3 | (Adachi et al., 2021) [32] | |
| Peff (effective jejuna permeability) | 5.31 | ADMET Predictor | |
| Blood to plasma ratio | 0.82 | (Administration, 2006) [33] | |
| k12(1/h) | 0.1289 | | PK plus |
| k21(1/h) | 0.0274 | | PK plus |
| v2(L/kg) | 12.86 | | PK plus |
